# Sources of adolescent mental disorder self-stigma and the effect of mental health on its formation: a moderated mediation model

**DOI:** 10.3389/fpsyg.2026.1759038

**Published:** 2026-05-25

**Authors:** Xi Wang, Haoran Niu, Hao Chen, Xin Ding, Xinyi Zhang

**Affiliations:** 1 School of Medicine and Health Management, Huazhong University of Science and Technology, Wuhan, China; 2 Social Security Department of Wuhan Finance Bureau, Wuhan, China; 3 Wuhan No.3 Hospital, Wuhan, China; 4 Hohhot Polytechnic University, Hohhot, China

**Keywords:** adolescent mental health, moderated mediation, peer stigma, self-stigma, stigma transformation, structural equation modeling

## Abstract

**Background:**

Adolescent mental disorders affect 14% of youth globally, yet treatment rates remain low (38%), particularly in low-resource settings. While stigma is a recognized barrier to care, limited evidence exists on how multi-level stigma (public, peer, family) transforms into self-stigmatization during adolescence—a critical developmental period characterized by heightened peer sensitivity.

**Methods:**

This cross-sectional study utilized a dyadic design to examine the transmission of stigma from caregivers to adolescents. We distributed survey sets to 8,500 dyads in Shandong, China. After matching and data cleaning, 4,702 valid adolescent-primary caregiver dyads remained. Validated instruments measured public stigma (STIG-9), peer stigma (PMHSS-R), family stigma (PDD), self-stigma (ISMI-10), and mental health status (GHQ-12).

**Results:**

Public stigma directly predicted peer stigma (*β* = 0.784 [95% CI: 0.752–0.816]), family stigma (*β* = 0.348 [95% CI: 0.313–0.383]), and self-stigma (*β* = 0.390 [95% CI: 0.351–0.429]). Peer stigma mediated public-to-self-stigma transmission (*β* = 0.426 [95% CI: 0.388–0.464]), while family stigma showed no direct effect. Adolescents with mental health problems exhibited higher baseline self-stigma (mean difference: 3.21 [95% CI: 2.83–3.59]) but attenuated response to rising peer stigma. Conversely, mentally healthy youth demonstrated accelerated self-stigmatization when peer stigma increased. Mental health significantly moderated public-to-family (*β* = −0.269 [95% CI: −0.309 to −0.229]) and peer-to-self-stigma pathways (*β* = −0.246 [95% CI: −0.287 to −0.205]).

**Conclusion:**

Peer stigma operates as the primary conduit through which societal stigma becomes internalized during adolescence, whereas family stigma plays a non-significant direct role. The paradoxical moderating effect of mental health—where affected youth show high baseline self-stigma but reduced reactivity—calls for developmentally tailored interventions. School-based peer initiatives should be prioritized alongside public awareness campaigns, while family programs should focus on support rather than stigma reduction.

## 1 Introduction

Mental disorders, a spectrum of clinically significant disturbance in one’s cognition, emotion or behaviors, are the rank among the top 10 leading causes of disease burden worldwide. As a unique and formative stage, adolescents are vulnerable to mental disorders and these diseases, in turn, threaten their short-term health (e.g., increased suicide) and long-term development (e.g., increased social isolation and reduced employment prospects; Gili et al., 2019; Angold et al., 1998; Cadman et al., 2012; Fergusson and Woodward, 2002; Wang et al., 2023; Hale and Viner, 2018). Globally, it is estimated that 14% of young people aged 10–19 years experience mental health conditions (World Health Organization, 2025), accounting for 15% of the total disease burden of this age group (World Health Organization, 2025). Early formal treatment can reduce the harm to adolescents caused by mental disorders (Hazell, 2007). However, a 2023 meta-analysis of 310,584 children and adolescents published in JAMA Network Open reported an overall treatment rate of 38% (95% CI, 30–45%) for any mental disorder (Wang et al., 2023). This situation is more severe in low- and middle-income countries (LMICs) due to limited access to health care services, poor mental health services infrastructure, and insufficient mental health budgets are compounded by stigma and low mental-health literacy, deterring young people and families from seeking care (Bolton et al., 2023; Villatoro et al., 2022).

Mental disorders stigma, defined as negative stereotypes, prejudicial attitudes, and discriminatory behaviors directed toward individuals with mental illness (Goffman, 2009; Erickson, 2006), is a major barrier for adolescent early formal healthcare seeking (Wang et al., 2023; Clement et al., 2015). On one hand, stigma makes adolescents with mental disorders feel ashamed, unconfident, and pessimistic by explicit and implicit rejection and discrimination from family, peers and society (Kaushik et al., 2016). On the other hand, adolescents may internalize stigma, called self-stigma, that further translated to reduced self-identify and self-devaluation (Hinshaw, 2005), in turn, resulting in less family and peer support and less likelihood for formal healthcare seeking (Chandra and Minkovitz, 2006). The vicious cycle significantly lowers adolescent probability for formal mental health services and results in worsen health outcomes. A 2020 US school-based study by Lindow et al. (Journal of Adolescent Health) demonstrated that higher self-stigma independently predicted significantly lower odds of seeking formal services (*β* = −0.28, *p* = 0.001), whereas lower stigma was associated with 11–15% more help-seeking contacts. These findings indicate that measurable stigma directly impedes adolescents’ utilization of mental healthcare services (Lindow et al., 2020).

To mitigate consequences of stigma on adolescent mental health, understanding its formation is crucial. However, the complexity of various sources of stigma and their interactions make it still veiled. As a distinct stage of limited independence and sensitivity to surrounding people attitudes (Lindow et al., 2020; Ragelienė, 2016; Samari et al., 2022), adolescents face various sources of mental health stigma, including family, peers, general populations and themselves (Adler and Wahl, 1998; Rose et al., 2007; Telesia et al., 2020). On the other hand, unlike adults, parents and peers often dominate adolescent social network (Elizalde-Resano et al., 2025; Liegghio, 2017) and they may serve as main sources of mental health prejudices and discrimination, leading the formation of self-stigma and less formal healthcare seeking (Corrigan and Rao, 2012).

One major barrier to early identification and proper treatment of adolescent mental disorders is stigma (Wang et al., 2023; Clement et al., 2015). Mental disorder stigma is commonly defined as negative stereotypes, prejudicial attitudes, and discriminatory behaviors directed toward individuals with mental illness (Goffman, 2009; Erickson, 2006). Adolescents with mental disorders often face social rejection and discrimination (Kaushik et al., 2016). Many of them also internalize these negative perceptions and behaviors, leading to self-stigma. They may feel ashamed, lose confidence, or develop a negative outlook on the future. On one hand, self-stigma can have lasting effects on adolescents’ sense of identity and autonomy (Hinshaw, 2005). On the other hand, it can also reduce their peer and social support. As a result, they may be less likely to seek help (Chandra and Minkovitz, 2006). This creates a harmful cycle that worsens their mental health.

Self-stigma related to mental disorders among adolescents exhibits distinct developmental characteristics. Adolescents are in a critical stage of identity formation, with limited psychological independence and heightened sensitivity to the attitudes of social attitudes (Lindow et al., 2020; Ragelienė, 2016; Samari et al., 2022). Compared to adults, adolescents have a stronger need for acceptance from peers and family members (Dailey, 2009; Roach, 2018). Recent studies show that adolescents with mental health problems face not only public stigma but also stigma from family members and peers (Adler and Wahl, 1998; Rose et al., 2007; Telesia et al., 2020). Parents and classmates often reflect the dominant social prejudices, which result in secondary labeling and the development of affiliate stigma toward adolescents (Elizalde-Resano et al., 2025; Liegghio, 2017). Repeated exposure to negative feedback can cause adolescents to internalize these attitudes. Over time, this process results in self-deprecating beliefs and negative self-perceptions (Corrigan and Rao, 2012). However, most existing research on youth stigma still focuses on public stigma. Few studies have systematically examined other sources of self-stigma among adolescents (Kaushik et al., 2016; Telesia et al., 2020). This gap may limit our understanding of the stigma they experience in daily life.

Although existing studies have emphasized the prevalence and multiple sources of self-stigma among adolescents, empirical research on the mechanisms underlying its transformation remains limited. Prior studies have shown that external stigma can become internalized by individuals with mental illness (Catalano et al., 2021). Earlier research on university students has also found that public stigma can transform into self-stigma (Vogel et al., 2013). However, such mechanisms have not been studied as extensively in adolescents as they have been in adults. Moreover, other studies indicate that adolescents with poorer mental health may be more sensitive to external stigma cues, which could, in turn, lead to higher levels of self-stigma (Law et al., 2007). In China, the age-standardized prevalence of mental disorders among adolescents was 8.9% in 2021. This rate represents roughly 2.8 million disability-adjusted life years (DALYs; Dong et al., 2025).

To address this gap, this study applies Structural Equation Modeling (SEM). SEM is well-suited to this research because it simultaneously analyzes complex variable relationships and accounts for measurement errors in latent constructs such as different types of stigma—capabilities that traditional regression analyses lack. Using survey data, we build an empirical model of adolescent self-stigma related to mental disorders to explore its origins and development. These insights will help design targeted anti-stigma strategies across society, schools, and families.

## 2 Methods

### 2.1 Theoretical framework

Stigma is a socially driven process that unfolds in multiple stages. Link and Phelan describe it as a result of social interactions that label someone as “abnormal” or “problematic.” These labels create and reinforce a divide between what society sees as “normal” and “abnormal” (Link et al., 1999). Many scholars agree that stigma develops through a sequence of cognition, attitude, and behavior (Burkholder et al., 1999; Heatherton, 2003). First, people form stereotypes about a group. These stereotypes turn into negative attitudes. Over time, they lead to discriminatory actions. Building on this foundation, Arjan and colleagues identify four sources of stigma: public, structural, affiliate, and self-stigma (Bos et al., 2013). Among adolescents, public stigma often appears through the words and actions of family members and peers. This public stigma can be enacted or perceived. If repeated, it may become internalized as self-stigma. Corrigan’s progressive model explains how this happens (Erickson, 2006; Corrigan and Rao, 2012). The process usually begins when a person notices how society views mental illness. This is the awareness stage. They may then agree with these public stereotypes. This is the agreement stage. After that, they may apply the same negative beliefs to themselves. This is the application stage. The result is often psychological harm. The person may lose confidence, feel ashamed, or believe they are less capable than others.

An adolescent’s mental health level can significantly influence their perception of stigma. Some studies suggest that adolescents who have experienced psychological distress tend to report lower levels of public stigma (Trompeter et al., 2022; Dardas et al., 2017). However, when it comes to self-stigma, the presence of mental health problems may actually intensify internalization (Law et al., 2007). These findings indicate that an adolescent’s mental health level may play a critical role in shaping how stigma is internalized.

**Figure 1:**
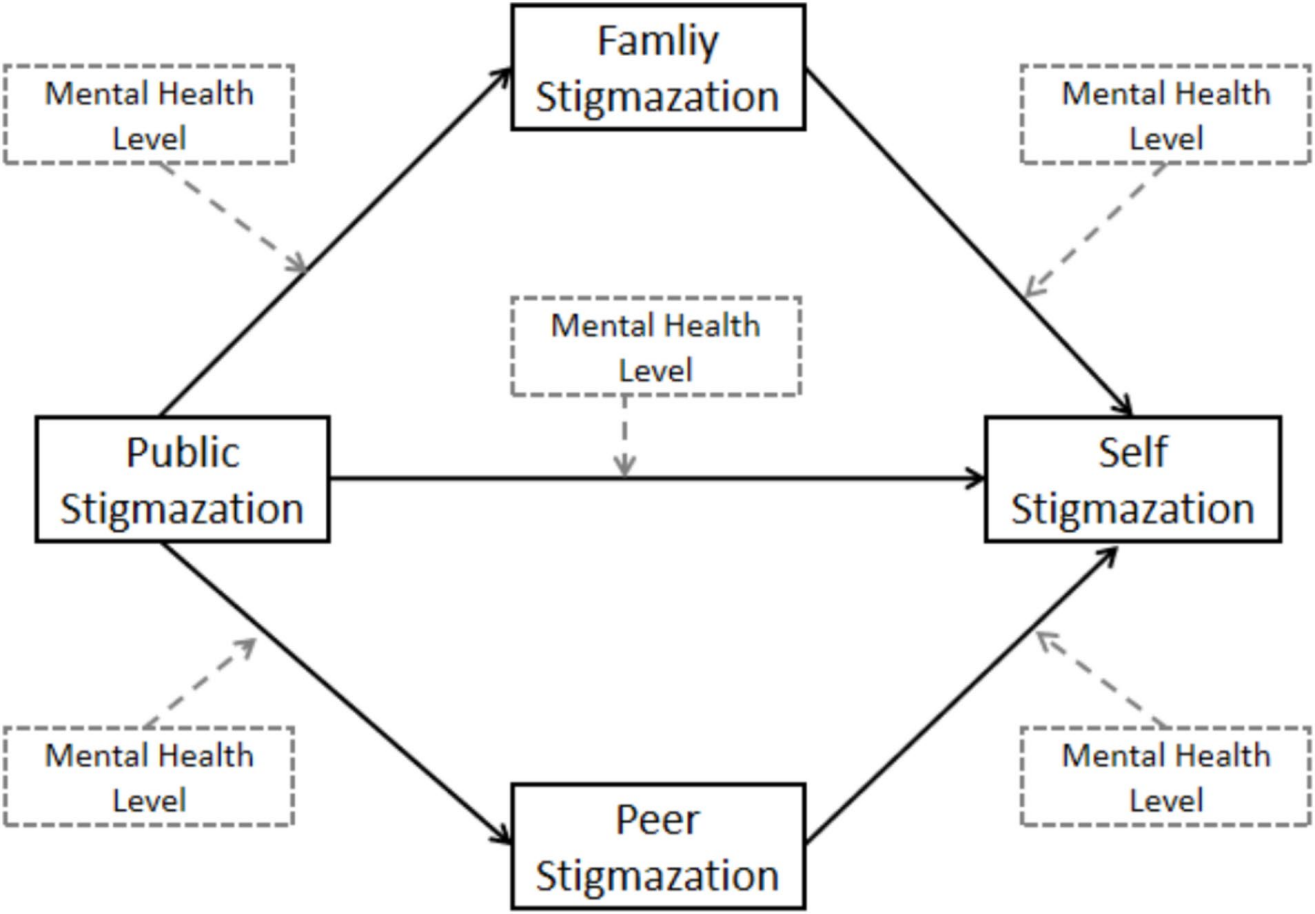
The hypothesized moderated mediation model.

On these grounds, Figure 1 presents our hypothesized moderated mediation model: we hypothesize that public stigma at the macro level drives group-based stigma among peers and family at the meso level, which in turn fosters self-stigma at the micro level. We further posit that mental health level moderates each of these pathways. By incorporating mental health as a moderator, our model captures how individual well-being shapes the dynamic internalization of external stigma during adolescence.

### 2.2 Design and setting

We adopted a cross-sectional survey design based on a cohort monitoring mental health conditions in a junior high school in Dongying, Shandong province, China. The age-standardized prevalence of mental disorders in Shandong ranks the top 5 among all provinces in China, 2021 (Dong et al., 2025), with over 20% adolescents experiencing depressive symptoms as well as anxiety and sleep disturbances (Xiu et al., 2022; Huang et al., 2025). Dongying is a prefecture city and its socio-economic development ranks … out of … among all cities in Shandong province. We selected a leading public junior high school in Dongying with a well-established mental health counseling center. There were three full-time counselors employed in the center and provide mental health services, including regular mental health extracurricular activities and group counseling sessions, mental health screening and routine mental health education. Despite these efforts, a 2023 pilot survey indicated 15% of students showed signs of mental health conditions, while the mental health services were seldomly used.

This study was conducted in Dongying City, Shandong Province, China, as part of a one-year prospective cohort. Shandong, a populous eastern coastal province with over 100 million residents, ranks among the top five provinces in age-standardized prevalence of mental disorders as of 2021 (Dong et al., 2025). Recent studies report that more than 20% of adolescents in the province experience depressive symptoms, along with common occurrences of anxiety and sleep disturbances (Xiu et al., 2022; Huang et al., 2025). Dongying, a major prefecture-level city in Shandong, was selected for this study. The participating school is the largest public junior high school in the city, enrolling approximately 11,000 students. It ranks among the top three in terms of educational resources, academic reputation, and psychological support infrastructure.

A longitudinal design was adopted, involving five waves of online psychological assessments at three-month intervals (September 2023 to September 2024). The empirical analysis focused on the second wave of the cohort, conducted in December 2023. We selected this wave because it achieved a stable participation rate and high data quality. At baseline, around 15% of students showed signs of psychological distress. In response, the school implemented various mental health initiatives, including the establishment of a counseling center, the employment of three full-time counselors, routine mental health screenings, and the maintenance of student psychological records. Additionally, the school conducts an annual Mental Health Education Month and offers regular mental health-themed extracurricular club activities and group counseling sessions.

### 2.3 Participants and sample

We used the entire sample of adolescents graded 6 to 8 in the participating junior high school (grade 9 was excluded since survey is not feasible due to adolescents’ full schedule of curriculum). We recruited both adolescents and their caregivers. Adolescents were eligible if they were (1) 12 to 18 years old; (2) capable to complete survey independently. Adolescent caregivers were eligible if they were (1) participating adolescent’s primary guardian (e.g., parents and grandparents); (2) capable to complete survey independently. Consents were obtained before survey from adolescents and their caregivers. Participants in this study were students in Grades 6 to 8 from an junior high school in Dongying City, Shandong Province, along with their primary caregivers. Eligible students met the following inclusion criteria: (1) aged between 12 and 18 years; (2) currently enrolled in junior high school; (3) willing to participate and capable of completing the questionnaire independently or with assistance from trained investigators. Eligible caregivers were defined as the students’ primary guardians who expressed willingness to participate and could complete the questionnaire independently or with support. Participants were excluded if either the student or the caregiver declined to participate or were uncooperative during data collection. The required sample size was calculated based on Jackson’s N:q rule for maximum likelihood estimation, adopting a conservative ratio of 20:1 (Jackson, 2003). Given an anticipated 20 model parameters in the structural equation model, a minimum of 400 participants was determined to be necessary to ensure sufficient statistical power.

### 2.4 Instruments

We developed instruments to assess mental health related stigma for adolescent and their caregivers based on existing scales. For adolescents, public, peer and self-stigma were collected. For their caregivers, public and family stigma were collected (table S…).

Public stigma was measured based on 9-item Stigma Scale (STIG-9; Gierk et al., 2018), which assessed people perceived negative societal beliefs, feelings, and behaviors toward mental disorders people (e.g., “I think most people think badly of someone who has been treated for a mental illness”). Peer stigma was assessed using Peer Mental Health Stigma Scale - revised version (PMHSS-R; Nearchou et al., 2021), in which adolescents peer agreement and awareness of stigma toward mental health was evaluated (e.g., “I believe that teenagers with emotional or behavioral problems are not as trustworthy as other teenagers”). Family stigma was evaluated using revised Chinese version of Perceived Devaluation-Discrimination Scale (PDD) that measured mental health stigma at the family level (e.g., “most people believe that entering a psychiatric hospital is a sign of personal failure”). Self-stigma was measured using the 10-item internalized stigma of mental illness scale (ISMI-10; Hammer and Toland, 2017; van Beukering et al., 2022), in which adolescent awareness, internalized shame and their agreement of negative stereotypes, prejudice and discrimination associated with mental illness were assessed.

**Public Stigma.** Public stigma was assessed using the 9-item Stigma Scale (STIG-9), which evaluates internalized shame that individuals may experience due to their minority group identity (Gierk et al., 2018). Items are rated on a 5-point Likert scale ranging from 1 to 5, with total scores on the public stigma subscale ranging from 9 to 45. The scale has demonstrated good psychometric properties in Chinese populations (Cronbach’s *α* = 0.86; Gierk et al., 2018).

**Peer Stigma.** Peer stigma was assessed using the Peer Mental Health Stigmatization Scale (PMHSS-R) developed by Nearchou et al. (2021), which measures adolescents’ awareness of and agreement with stigmatizing attitudes toward mental health problems among peers (e.g., “I believe that teenagers with emotional or behavioral problems are not as trustworthy as other teenagers”). The PMHSS-R has demonstrated adequate psychometric properties, with Cronbach’s *α* = 0.79 for the Stigma Awareness subscale and α = 0.72 for the Stigma Agreement subscale (Nearchou et al., 2021). Items are rated on a 5-point Likert scale.

**Family Stigma.** Family stigma of mental health was assessed using the revised Chinese version of the Perceived Devaluation-Discrimination Scale (PDD), which measures the stigma associated with mental illness at the family level. Each item is rated on a 5-point Likert scale, and the total score ranges from 12 to 60. The original PDD was developed by Link (1987) and has demonstrated acceptable reliability across diverse cultural contexts (Cronbach’s α = 0.76–0.88). The Chinese version has shown adequate reliability and validity in Chinese populations.

**Self-Stigma.** Adolescent self-stigma related to mental health (Self-Stigma, SS) was assessed using the brief 10-item version of the Internalized Stigma of Mental Illness Scale (ISMI-10; Boyd et al., 2014). It is suitable for assessing personal or internalized shame associated with mental illness, as well as individuals’ awareness, agreement with, and application of negative stereotypes, prejudice, and discrimination (Hammer and Toland, 2017; van Beukering et al., 2022). The ISMI-10 was validated by Hammer and Toland (2017) and has demonstrated good internal consistency (Cronbach’s α = 0.75) and construct validity among individuals with depression. Further validation by van Beukering et al. (2022) confirmed its reliability (Cronbach’s α = 0.83) in a Dutch sample. Items are rated on a 4-point Likert scale ranging from 1 (strongly disagree) to 4 (strongly agree), with total scores ranging from 10 to 40. All items were answered based on a 5-likert scale and a higher score indicated increased stigma related to mental health.

**Mental Health Level.** Mental health was assessed with the Chinese version of the General Health Questionnaire (GHQ-12), a widely used screening tool for psychological distress and common mental disorders. The GHQ-12 has demonstrated strong cross-cultural validity and internal consistency (Cronbach’s α = 0.82–0.86) across diverse populations (Goldberg et al., 1997), and the Chinese version validated by Chong and Wilkinson (1989) has shown adequate reliability and validity in Chinese populations. Each item is rated on a 4-point response scale, with total scores ranging from 0 to 36. Higher scores indicate greater psychological distress. All instruments utilized in this study are open-access for academic research or were used with necessary institutional permissions. Validated Chinese versions were used for all scales.

### 2.5 Data collection

Data for this study were collected through an online survey administered via the Wenjuanxing platform, a widely used digital questionnaire tool in China. Only responses from successfully matched student–caregiver pairs were retained for statistical analysis. Prior to the main survey, a pilot study was conducted involving 10 student–caregiver pairs recruited through convenience sampling. Feedback from the pilot phase was used to refine the structure, wording, and clarity of the questionnaire. The revised version was finalized and distributed electronically through Wenjuanxing during the formal data collection phase.

To ensure data integrity, several quality control procedures were implemented. First, the participating school coordinated and supervised the administration of each survey wave. Homeroom teachers received standardized training prior to data collection. Each wave lasted 2 weeks, with interim data checks and reminder notifications issued to classes with low response rates. Second, the online platform incorporated built-in quality controls, including minimum page completion times derived from pilot testing, input validation mechanisms, and clarifying instructions for items prone to misinterpretation. Third, to minimize participant attrition, surveys were scheduled during homeroom periods or weekends in coordination with school staff, reducing conflicts with academic schedules. Fourth, invalid responses were excluded based on predefined criteria, including format errors, missing codes, illogical age entries, patterned responses, duplicate submissions, and unmatched student–caregiver records.

The sampling flow was as follows: we initially distributed survey sets to 8,500 student caregiver dyads. A total of 14,666 individual questionnaires were received (7,731 from students and 6,935 from caregivers). After excluding invalid responses based on predefined criteria (e.g., patterned responses, completion time < 300 s, or illogical age entries), 9,952 valid individual questionnaires remained. Finally, by matching student and caregiver IDs, the analytical sample was narrowed to 4,702 complete and valid student parent dyads (55.3% dyad completion rate). In instances where more than one parent participated for the same student, the mean value of the parental responses was assigned to the student record.

### 2.6 Statistical analysis

The collected questionnaire data were cleaned and coded prior to analysis. The characteristics of participants were described using frequency distributions for categorical variables and means (s.d) for continuous variables. For the GHQ-12, total scores were calculated after reverse-coding negatively worded items, and a cut-off value of 27 was applied to dichotomize mental health status (Goldberg et al., 1997; Chong and Wilkinson, 1989). Participants scoring above 27 were coded as 1 (poor mental health), and those scoring 27 or below were coded as 0. Stigma-related items were scored on a 5-point Likert scale. Negatively worded items were reverse-coded to ensure that higher scores consistently reflected higher levels of perceived stigma.

We employed structural equation modeling (SEM) to test our hypotheses regarding the transformation mechanisms of stigmatization. We also added moderated mediation analyses for each path of this standard model. All data analyses were conducted using Mplus 8.7, and the interaction effect plots were generated using Python 8.3. The data analysis was carried out in two steps. First, based on the inclusion of control variables, we tested the validity of the structural equation model capturing the transmission relationships of stigmatization—this constitutes the standard structural equation model. Then, we introduced the moderating effect of mental health levels into different paths of the standard SEM.

Furthermore, we extracted coefficients and intercepts of stigmatization variables from the significant interaction paths and fixed the independent variable scores in the moderated mediation model at one standard deviation above and below the mean. Using the spotlight analysis approach, we more concretely illustrated the moderating effect of mental health issues on the transformation model of stigmatization.

## 3 Results

### 3.1 Characteristics of respondents

The demographic characteristics of the 4,702 respondents are summarized in Table 1. The sample had a mean age of 12.464 years (SD = 0.94), and 53% were female. The majority of respondents came from two-parent households with siblings. Household income was predominantly concentrated in middle-income brackets, and chronic disease prevalence was low. Most participants reported good physical health.

**Table 1:** Characteristics of respondents.

Characteristics	Mean ± SD^*^/N (%)
Age (years)	12.46 ± 0.94
Sex	
Male	2,210(47)
Female	2,492(53)
Only child	
No	3,527(75.01)
Yes	1,175(24.99)
Primary caregiver	
Mother	979(20.82)
Father	106(2.25)
Both parents	3,494(74.31)
Others	123(2.62)
Family household size	4.02 **±** 1.06
Annual household income (CNY^*^, ¥)	
Below 80,000	1,077(22.91)
80,000–140,000	1,475(31.37)
140,000–260,000	1,509(32.08)
Above 260,000-	641(13.63)
Chronic disease	
No	4,652(98.94)
Yes	50(1.06)
Know someone with mental health issues	
No	4,094(87.07)
Yes	608(12.93)
Experienced negative events in the past year	
No	2,398(51)
Yes	2,304(49)
Self-rated physical health	
Very Poor	10(0.21)
Poor	38(0.81)
Fair	315(6.70)
Good	1997(42.47)
Excellent	2,342(49.81)
Mental health level	21.04 ± 6.05

*SD, Standard Deviation. CNY, Chinese Yuan.

### 3.2 Features of each dimension in the stigma transformation mechanism model: an analytical approach

In terms constructs based on theoretical Model of the transformation mechanisms of stigmatization (Table 2), regarding stigma levels, respondents indicated that family stigma was the highest (mean = 27.2, SD = 5.05), followed by peer stigma (mean = 23.15, SD = 6.96) and public stigma (mean = 22.61, SD = 7.37), while self-stigma was the lowest (mean = 17.06, SD = 5.02). In addition, Kruskal-Wallis H test results showed that students with poor mental health had significantly higher levels of public stigma (*p* = 0.001), peer stigma (p = 0.001), family stigma (*p* = 0.0068), and self-stigma (*p* = 0.0001) compared to all students.

**Table 2:** Respondents on various dimensions of stigma.

Measurement	Score		
	Mental health (mean ± SD)	Mental Ill-health (mean ± SD)	Total (mean ± SD)
Self-stigma	16.44 ± 4.89	19.65 ± 4.73	17.06 ± 5.02
Public stigma	22.17 ± 7.32	24.43 ± 7.30	22.61 ± 7.37
Family stigma	27.08 ± 5.15	27.71 ± 4.62	27.20 ± 5.05
Peer stigma	22.71 ± 6.78	24.96 ± 7.39	23.15 ± 6.96

### 3.3 SEM of the transformation of mental health stigma

**Figure 2:**
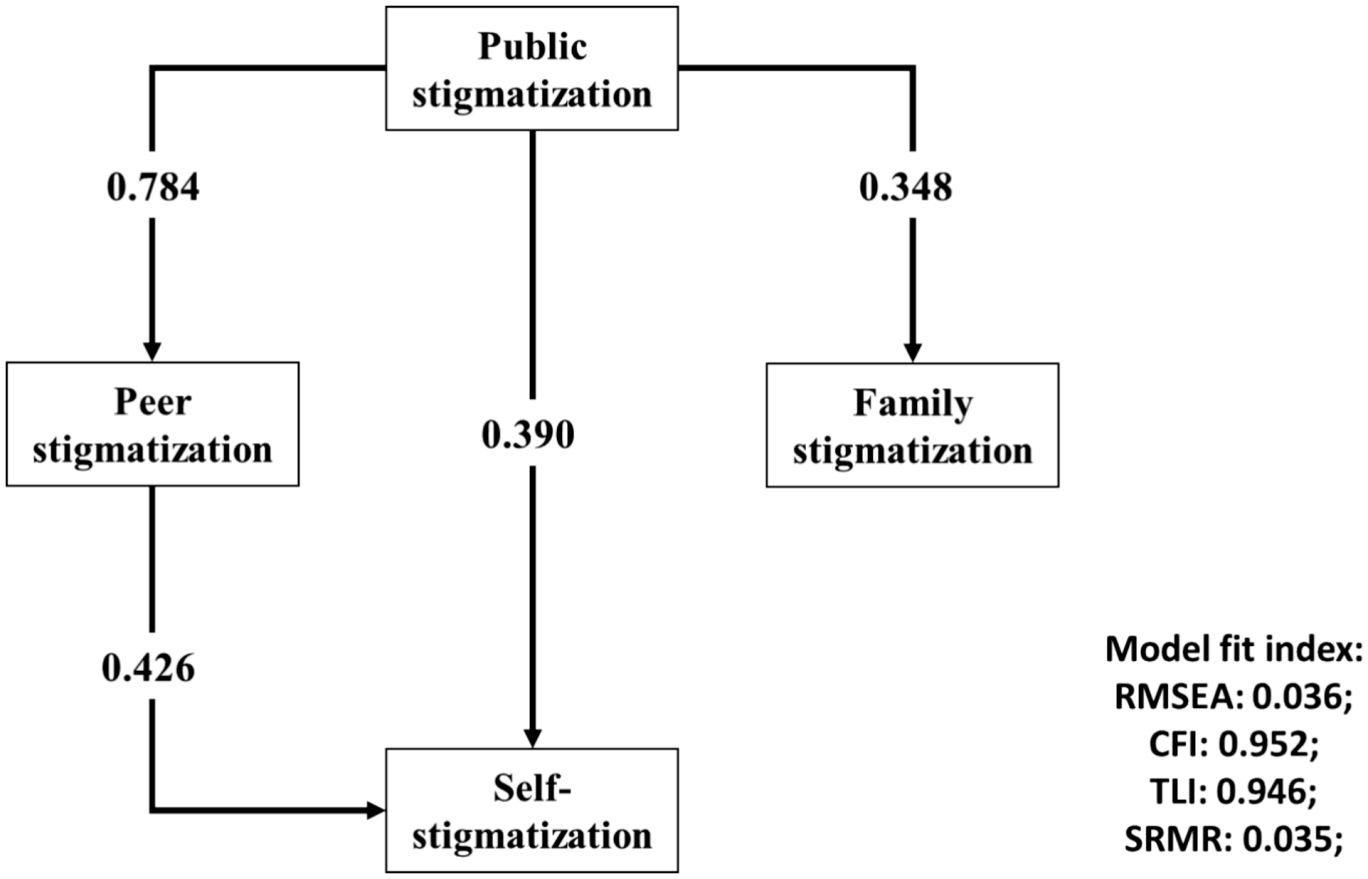
The transformation of mental health stigma. Only significant pathways (*p* < 0.05) were reported with standardized path coefficients.

The SEM confirmed that the survey data fitted stigmatization transformation model well (Figure 2), with all fitness index met the criteria (RMSEA = 0.036, 90% CI: 0.035–0.037, CFI = 0.952, TLI = 0.946, SRMR = 0.035).

According to the results of SEM, public stigmatization exerted significant positive effects on peer stigmatization (*β* = 0.784, *p* < 0.001), family stigmatization (*β* = 0.348, *p* < 0.001), and self-stigmatization (*β* = 0.390, *p* < 0.001), indicating that all other forms of stigmatization increased in parallel with elevated public stigmatization levels. Additionally, peer stigmatization demonstrated a significant positive influence on self-stigmatization (*β* = 0.426, *p* < 0.001), suggesting that higher peer stigmatization was associated with heightened self-stigmatization.

### 3.4 A model for transforming stigma in mental health issues regulation

**Figure 3:**
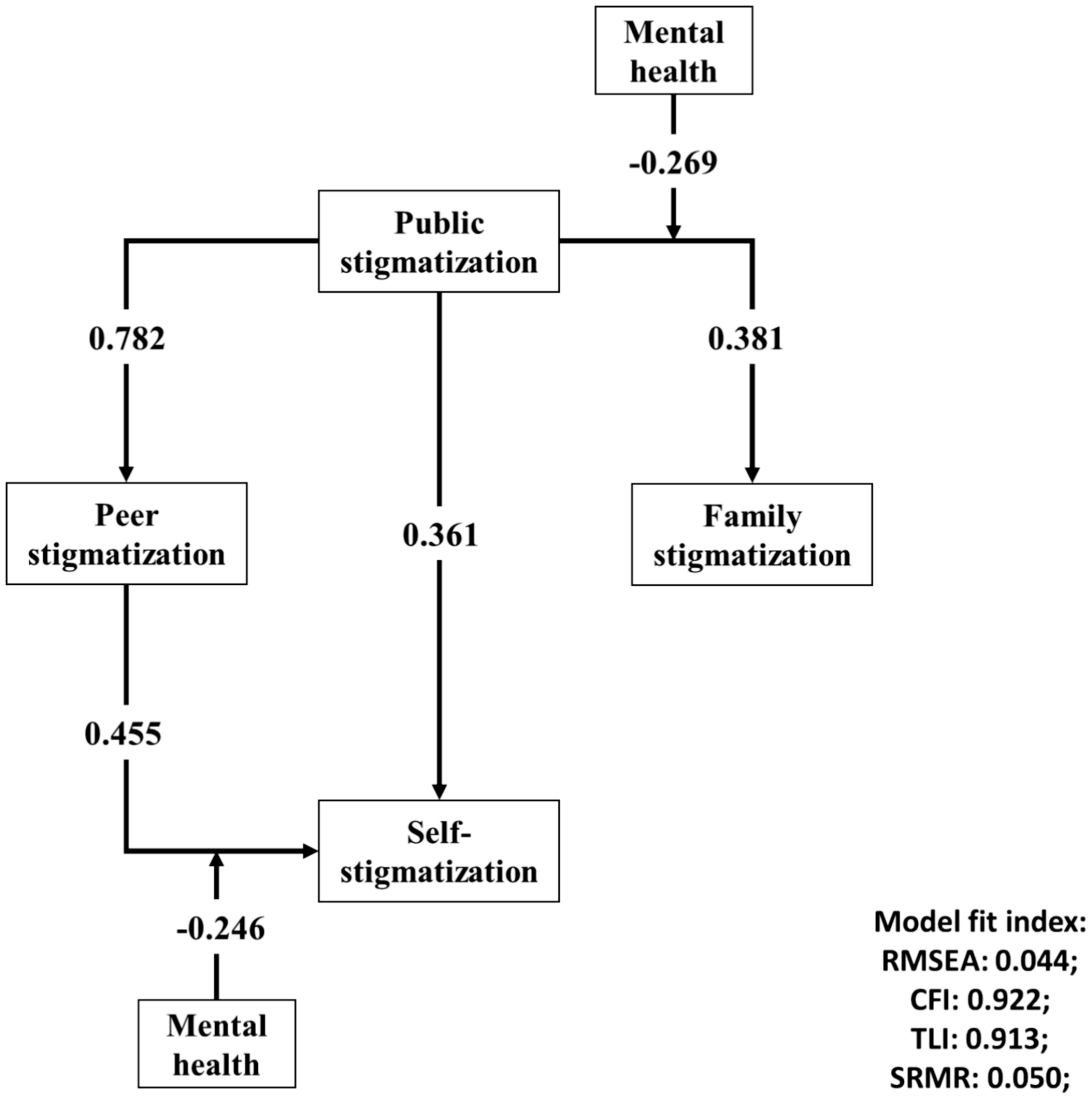
The transformation of mental health stigma moderated by mental health level. Only significant pathways (*p* < 0.05) were reported with standardized path coefficients.

The moderation model fits well with the moderating effect of mental health problems on the transformation of mental health stigmatization (Figure 3), with all fitness index met the criteria (RMSEA = 0.044, 90% CI: 0.043–0.044, CFI = 0.922, TLI = 0.913, SRMR = 0.050).

In the moderated model, the transmission pathways of stigmatization remain fundamentally consistent with the standard structural equation model, differing only in the standardized coefficients. Notably, mental health issues exhibit significant inhibitory effects: dampening the conversion from public stigmatization to family stigmatization (*β* = −0.269, *p* < 0.001) and suppressing the conversion from peer stigmatization to self-stigmatization (*β* = −0.246, *p* < 0.001).

### 3.5 Moderating effects of stigmatization under different levels of mental health

**Figure 4:**
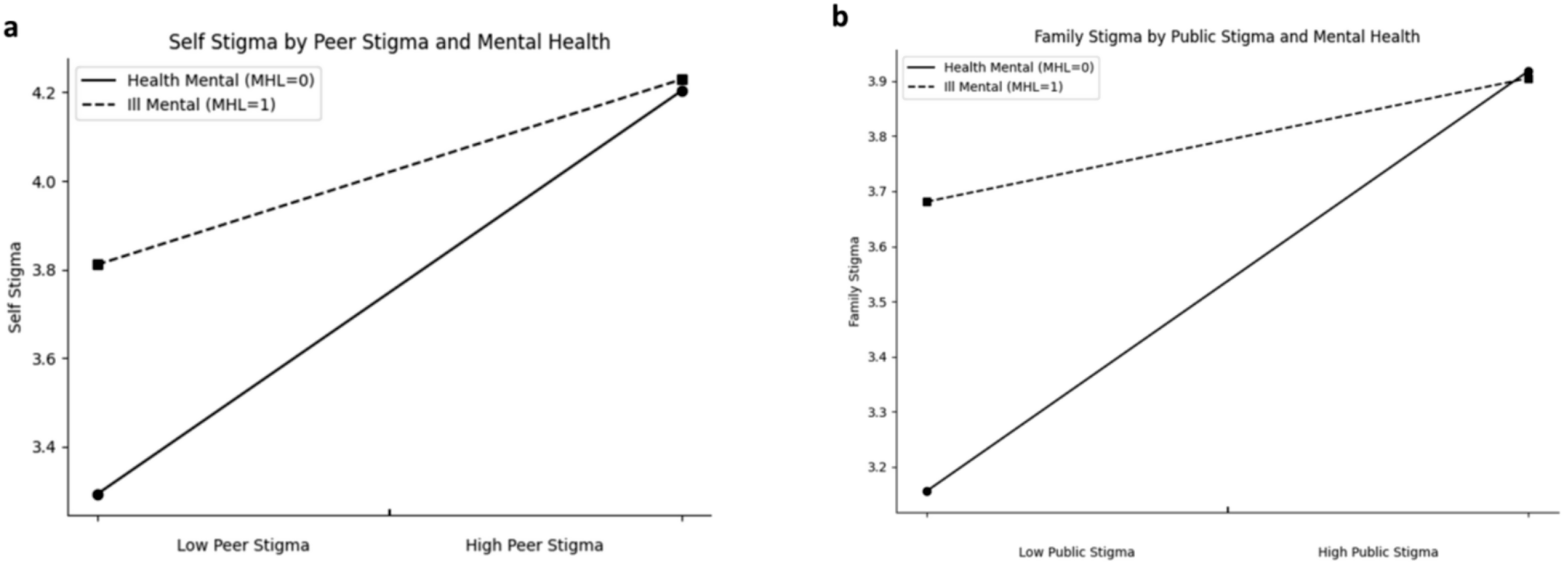
Moderating effects of mental health level on stigma transformation pathways. **(a)** Self-stigmatization by peer stigmatization and mental health level. Solid line: healthy mental group (MHL = 0); dashed line: poor mental group (MHL = 1). **(b)** Family stigmatization by public stigmatization and mental health level. Solid line: healthy mental group (MHL = 0); dashed line: poor mental group (MHL = 1).

We used interaction effect plots to illustrate the moderating effects of peer stigmatization and mental health level on self-stigmatization (Figure 4), as well as the moderating effects of public stigmatization and mental health level on family stigmatization (Figure 4).

In Figure 4, the level of self-stigmatization among adolescents with poor mental health is higher than that among those with good mental health. However, compared to adolescents with poor mental health, the self-stigmatization of those with good mental health increases more rapidly as peer stigmatization increases.

Similarly, in the case of family stigmatization (Figure 4), adolescents with poor mental health generally exhibit higher levels of family stigmatization than those with good mental health. Yet, compared to adolescents with poor mental health, the family stigmatization of those with good mental health increases more rapidly as public stigmatization increases.

## 4 Discussion

To the best of our knowledge, this is the first study to offer quantitative evidence on the sources and associative pathways of self-stigma among adolescents. The results show a clear pattern. Our findings reveal a clear associative pathways: external stigma ultimately becomes internalized as self-stigma in adolescents. Specifically, higher levels of public stigma are associated with stronger perceptions of group stigma, particularly within peer groups. In turn, peer stigma significantly contributes to the development of adolescent self-stigma. However, stigma from family members did not directly lead to self-stigma in our data. We also found that mental health plays a role in this process. Adolescents with better mental health showed a sharper rise in group and self-stigma when external stigma increased. In contrast, those with poorer mental health already had high levels of self-stigma, even when external stigma was low.

Our study shows that public and peer stigma are the main sources of self-stigma among adolescents. This provides further empirical support for Corrigan’s progressive model of self-stigma (Corrigan and Rao, 2012; Goepfert et al., 2019). According to the model, when adolescents with mental health problems are repeatedly exposed to negative attitudes and social rejection from the public or peers, they may start to accept these views. Over time, they may believe the stereotypes and apply them to themselves, resulting in harmful self-perceptions. Adolescence is a critical period for identity formation and value development (Hinshaw, 2005; Elizalde-Resano et al., 2025; DeLuca, 2020). Guided by Erikson’s theory of identity formation, this stage is characterized by a significant shift toward peer affiliation and a heightened sensitivity to social feedback. This is further explained by Elkind’s concept of the “imaginary audience,” where adolescents perceive themselves as being constantly scrutinized by their peers, making peer-delivered stigma particularly influential on their developing self-concept. Research in developmental psychology shows that adolescents are highly sensitive to peer opinions and social acceptance (J. Clin. Med., 2025). In many cases, this sensitivity exceeds their responsiveness to family views. Our findings are consistent with this pattern. Previous studies also show that while fewer than half of adolescents report stigma from family members, stigma from peers is much more common (Elizalde-Resano et al., 2025).

Interestingly, this study found no significant direct effect of family stigma on adolescents’ self-stigma. This contrasts with some previous research suggesting that parental attitudes and parents’ own stigma experiences may increase adolescents’ risk of internalized stigma (Giannopoulou et al., 2025; Dikeç et al., 2022). Several factors may explain this finding. First, developmental research shows that adolescents are more sensitive to peer opinions (DeLuca, 2020). During this stage, peer influence often outweighs parental influence on self-identity. Second, family stigma may not be easily perceived or internalized by adolescents. In cultures that value family harmony and social image, parents may express protection and care, even if they feel shame. Some parents may also hide the illness or avoid discussing it, reducing adolescents’ exposure to stigma (Samari et al., 2022; Goepfert et al., 2019). Third, the effect of family stigma may be indirect. It may reduce parental support for help-seeking or increase family stress, but these effects may not directly lead to self-stigma from the adolescent’s perspective (Moses, 2014). Finally, strong family support and good parent–child relationships may help protect adolescents from internalizing stigma. These positive factors may build psychological resilience even in a stigmatizing environment.

In the digital era, the influence of social media on adolescent self-concept cannot be overlooked, as these platforms play a central role in contemporary identity development and peer relationships (Ragelienė, 2016). Social media platforms function as a modern, pervasive “imaginary audience,” where negative labeling and mental health stereotypes can be amplified and disseminated rapidly (Rose et al., 2007). This “cyber-stigma” may act as a digitized extension of peer stigma, potentially accelerating the internalization process by reinforcing public conceptions of mental illness (Link et al., 1999). Given that adolescents spend significant time in online social spaces, the digital echo chamber may intensify their sensitivity to societal prejudices, further fueling the formation of self-stigma.

Our study shows that mental health level moderates how adolescents internalize stigma. We found that adolescents with poorer mental health often show high levels of self-stigma from the beginning. Even when peer stigma is low, they tend to hold negative self-beliefs and feel shame. This suggests that poor mental health increases stigma sensitivity and awareness (Kaushik et al., 2016; Elizalde-Resano et al., 2025). Surprisingly, we also observed that as peer stigma rose, self-stigma increased more sharply among adolescents with better mental health. Those with poor mental health may have already reached high levels of self-stigma, leaving less room for further increase. This pattern suggests a potential desensitization or ego-defense mechanism, where adolescents with significant psychological distress “cap” their internalized stigma to shield their remaining self-identity. In contrast, adolescents with better mental health started lower and had more space for self-stigma to grow. Previous studies (An et al., 2020; Nursalina et al., 2026) suggest that some adolescents develop coping or recovery strategies after facing mental health challenges. These may help reduce or stabilize self-stigma in the short term. This differs from findings in adult samples, where self-stigma usually increases steadily with external stigma, regardless of mental health (Vogel et al., 2006; Livingston and Boyd, 2010). These findings highlight the need for tailored interventions. Mentally healthy adolescents may benefit from resilience training, while those with high self-stigma require early and targeted support.

This study has several strengths. First, we systematically assessed stigma at public, group (peer/family), and individual levels, and examined how self-stigma formats from these layers. Second, the analysis was based on a large sample from a mental health screening cohort, which enhanced the robustness and credibility of the findings.

Several limitations should also be acknowledged. First, the cross-sectional design limits our ability to confirm causal directions or track changes over time. Therefore, the pathways identified in this study should be interpreted as associations rather than longitudinal causality. Still, the use of a theory-driven model and structural equation modeling helped support causal interpretation. Second, although the sample was large (4,702 student–guardian pairs) and diverse in terms of gender, grade level, and family background, and diverse, generalizability to other regions or cultural settings requires further testing. Third, as participation was voluntary, selection bias may exist. However, we applied quality control measures and adjusted for key demographic variables. Fourth, as stigma is a sensitive topic, self-reports may be affected by social desirability or recall bias. Some family stigma may also go unnoticed by adolescents. Future studies should use multi-wave longitudinal designs and draw from culturally and geographically diverse populations to further validate and expand the transformation model of adolescent self-stigma proposed in this study.

Our findings highlight the need for coordinated anti-stigma efforts across schools, families, and the broader community. First, schools play a key role. Mental health education, peer-led activities, and contact-based programs with recovered individuals can help reduce peer stigma, a major driver of adolescent self-stigma. Interventions should specifically target the reduction of cyber-stigma within digital social networks Second, public campaigns and media initiatives should target negative societal stereotypes. Changing these views can help curb both peer stigma and its potential spillover into families. Third, although family stigma did not directly predict self-stigma in our study, parental support remains essential. Parental education programs that offer accurate knowledge and coping strategies can help prevent societal prejudice from entering the home. Together, these strategies can disrupt the stigma cycle and encourage adolescents to seek help.

## Data Availability

The raw data supporting the conclusions of this article will be made available by the authors, without undue reservation.
